# PERIANAL BASAL CELL CARCINOMA—AN UNUSUAL SITE OF OCCURRENCE

**DOI:** 10.4103/0019-5154.62758

**Published:** 2010

**Authors:** D V Nagendra Naidu, V Rajakumar

**Affiliations:** *From the Department of General Surgery, Sundaram Medical Foundation, Chennai, India.*

**Keywords:** *Basal cell carcinoma*, *local excision*, *malignancy*, *perianal*

## Abstract

Basal cell carcinoma is the most common nonmelanoma skin cancer. Its occurrence in the perianal region is very rare. Awareness of its benign behavior in this region, in contrast to the earlier reports, is vital in its management. Local excision seems to provide adequate control. We are herewith reporting an extremely rare case of a 69-year-old male with basal cell carcinoma treated adequately with local excision.

## Introduction

Basal cell carcinoma (BCC) remains the most common nonmelanoma skin cancer.[1] Though its predilection for the head and neck (around 75-86%), due to solar exposure is well known, its occurrence on non-exposed areas such as perianal region is extremely rare. There are few anecdotal case reports and only three small case series in the western literature.[1–6] But the incidence of this rare entity has not been reported in the literature of this part of the Indian subcontinent.

The awareness of the benign behavior of BCC at the perianal site is of significance in its management, as earlier reports had suggested an aggressive behavior of this tumor in the perianal region unlike those arising in more usual areas.[7]

## Case Report

**Figure 1:**
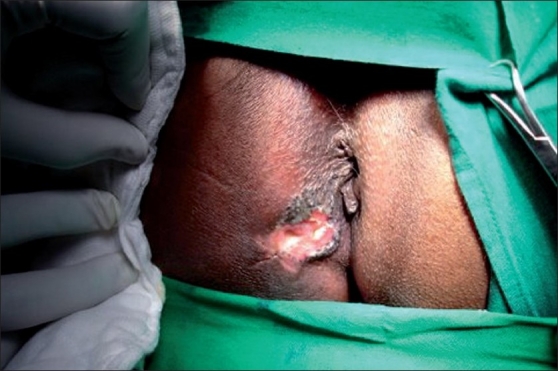
Shows pre-op picture of perianal basal cell carcinoma

A 69-year-old male presented with complaints of upper abdominal pain of 2 months duration with increased intensity for the past 1 week. He had history of constipation. He also complained of a painless ulcerative skin lesion in the perianal region, for the last 7 years. He was a known case of diabetic mellitus with no significant family history. On examination he was found to have an ulcer of the size 3cm × 2 cm in the perianal region between 7 to 9 ‘o clock position [Figure 1]. It was irregularly shaped, non-tender ulcer with hard raised edges, spreading hyper-pigmented margins and surrounding induration. Per-rectal and proctoscopic examinations were normal. He had history of applying ointment clotrimazole on and off over the same region. Rest of the systemic examination was unremarkable.

His hemogram and biochemical parameters were within normal limits. Other investigations for abdominal pain and constipation were done. Upper GI endoscopy revealed Grade 1 Reflux esophagitis. Ultrasonography revealed a small gall bladder calculus with normal wall thickness. Sigmoidoscopy was normal. Dermatologist opinion was sought for this skin lesion. Differential diagnoses considered at this point for the perianal lesion were tuberculous ulcer, severe candidial erosive ulcer, Crohn's disease, and fistula in ano. Negative history of symptoms or signs suggestive of Crohn's (such as painful ulcer, anal incontinence, bleeding) and fistula in ano along with normal rectal, proctoscopic and sigmoidoscopic examination ruled out the latter two. Subsequent to this evaluation, the patient was subjected to a wedge shaped biopsy of the lesion, including a part of the ulcer with its base and a part of the normal skin around it. Biopsy specimen was then sent for histopathology and also requesting for PAS stain, Gram stain and AFB stain and culture, which revealed no growth. Routine serology pre-operation is not done in our institution. In view of a reliable history taken to exclude any predisposition to sexually transmitted diseases due to altered sexual behavior in this patient, the serology investigation was withheld.

**Figure 2:**
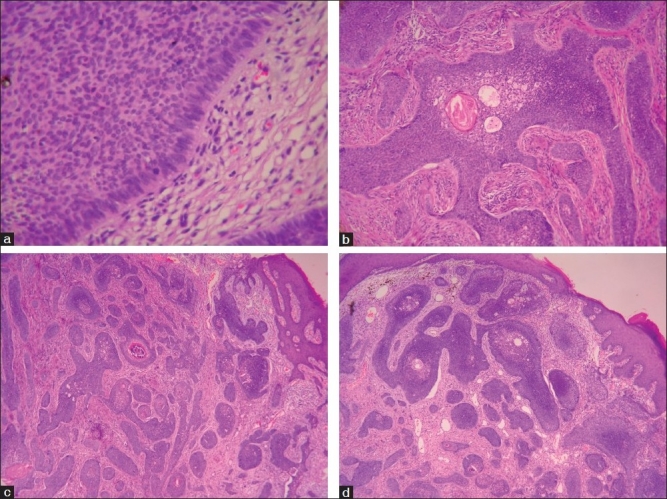
Cell clusters with peripheral palisading of nuclei characteristic of basal cell carcinoma

The biopsy section showed skin extensively being infiltrated by a tumor composed of nests of basaloid cells exhibiting increased mitotic activity. Centrally keratin pearl formation and areas of necrosis were also seen showing focal calcification. Cell clusters showed peripheral palisading of nuclei. There was much ulceration covered by neutrophillic exudates. The lesion did not infiltrate the underlying adipose tissue [Figure 2]. Deep margins were negative. Medical oncologist opinion was sought at this junction and a consensus was formed for a wide local excision of the ulcerative lesion. Intraoperatively, the lesion was found to be 0.5 cm in depth and a wide local excision was done. Specimen histopathology confirmed the previous report with involvement of posterior margins on the surface. Repeat excision was done with confirmation of a clear margin in the final HPE report.

Postoperative recovery was uneventful. No adjuvant radiotherapy or chemotherapy was given as suggested by the medical oncologist. The patient has been on regular follow up for the last 8 months. There has been no recurrence.

## Discussion

BCC occurs commonly on sun-exposed cutaneous surfaces. Its occurrence in the perianal region remains extremely rare. Only few case reports and three small case series have been reported in the Western literature, but none so far in the literature, in this part of the globe.

Earlier studies have reported its incidence to be on the higher side in the middle-aged to elderly patients. Around 30% of these lesions were ulcerated, high-lighting the possible delay in diagnosis. Patients often delayed seeking treatment for what they considered trivial itching or simple irritation, or physicians initially missed these cancers for inflammatory or infectious dermatoses as seen in our patient's history.[8] Due to the innocuous appearance of BCC at these sites, it is recommended that a biopsy of all suspect lesions be performed.

Histological verification of lesions appearing as BCC in the perianal region is warranted, to identify those with more aggressive histological subtypes and to distinguish them from cloacogenic carcinoma. Cloacogenic carcinoma or the more highly malignant basaloid small cell carcinomas occur more often near the dentate line or within the anal canal. These tumors behave biologically aggressive, metastasize rapidly, and have worse prognosis,[1] as compared to BCC located in the perianal region, which behave like their counterparts on sun-exposed skin.

Although causative factors, such as ultraviolet light, pre-existing skin conditions, and genetics, have been implicated in the development of BCCs on sun-exposed skin, the cause of these lesions in the perianal area is unknown. Earlier reports do implicate radiotherapy to the pelvic region,[7] chronic skin irritations as seen with chronic pruritus vulvae or ani, depressed immune surveillance due to ultraviolet radiation in the pathogenesis of perinanal BCC.

Treatment modalities including wide local excision, electrodessication and curettage and Mohs micrographic surgery have been reported in the world literature. True basal cell epitheliomas, being nonaggressive lesions, seem adequately treated by local excision to clear margins with no evidences of either local recurrence or distant metastasis seen during the follow-up period.[1] Alertness to the rare occurrence of this tumor at perianal site with understanding of its clinical course can thus prevent delay in its diagnosis and morbid aggressiveness in the management of the disease.
